# Diagnostic Value of Two-Dimensional Transvaginal Ultrasound Combined with Contrast-Enhanced Ultrasound in Ovarian Cancer

**DOI:** 10.3389/fsurg.2022.898365

**Published:** 2022-05-27

**Authors:** Rong Hu, Gulina Shahai, Hui Liu, Yuling Feng, Hong Xiang

**Affiliations:** Department of Ultrasound in Obstetrics and Gynecology, Key Laboratory of Ultrasound Medicine of Xinjiang, The First Affiliated Hospital of Xinjiang Medical University, Urumqi, Xinjiang, China

**Keywords:** ovarian cancer, vagina, two-dimensional ultrasound, contrast-enhanced ultrasound, diagnostic value

## Abstract

**Objective:**

Explore the value of two-dimensional transvaginal ultrasound combined with contrast-enhanced ultrasound in the differential diagnosis of ovarian cancer, so as to provide the basis for clinical diagnosis and treatment of ovarian cancer.

**Methods:**

A total of 100 suspected ovarian cancer patients who were admitted to our hospital from January 2019 to December 2021 were selected as the research subjects, including 62 ovarian cancer patients (ovarian cancer group) and 38 ovarian benign tumor patients (benign group). Two-dimensional vaginal ultrasound and contrast-enhanced ultrasound were performed in both groups. The differences in PI, RI, EDV, PSV, and VM parameters of the two groups as well as those of patients with ovarian cancer of different grades were compared. Record the contrast-enhanced ultrasound parameters such as AT, TTP and IMAX, and determine the diagnostic value.

**Results:**

The PI and RI of the ovarian cancer group were lower than those of the benign ovarian tumor group, and the EDV, PSV and VM of the ovarian cancer group were higher than those of the benign ovarian tumor group (*p *< 0.05). The PI and RI of the patients in stage I–II of the ovarian cancer group were higher than those in stage III–IV, and the EDV, PSV and VM were lower than those in the patients in stage III–IV, with statistical significance (*p *< 0.05). The results of contrast-enhanced ultrasound showed that the AT and TTP values in the ovarian cancer group were significantly shorter than those in the benign group, and the peak intensity was significantly higher than that in the benign group, and the differences were statistically significant (*p *< 0.05). The sensitivity, specificity, positive predictive value, negative predictive value and accuracy of two-dimensional ultrasound combined with contrast-enhanced ultrasound in the diagnosis of ovarian cancer were high, 95.16%(59/62), 86.84%(33/38), 92.19%(59/64), 91.67%(33/36) and 92.00%(92/100), respectively.

**Conclusion:**

Contrast-enhanced ultrasound to some extent makes up for the deficiencies of conventional ultrasound, is helpful to detect early ovarian cancer, and can be used for the differential diagnosis of small ovarian tumors with difficult two-dimensional ultrasound diagnosis. Two-dimensional ultrasound combined with contrast-enhanced ultrasound can effectively improve the detection rate and differential diagnosis value of ovarian cancer, which is of great significance in the early diagnosis and differentiation of ovarian cancer.

## Introduction

Ovarian cancer is a common malignant tumor in the reproductive system of women. In recent years, the incidence rate has increased year by year, and the mortality rate is high, which poses a serious threat to the physical and mental health of women (1). The early symptoms of ovarian cancer are hidden, and most patients are in the advanced stage when the diagnosis is confirmed, with high malignancy and poor treatment effect. In addition, the prognosis of ovarian cancer among all gynecological malignant tumors is the worst (2). Therefore, early diagnosis and early standardized treatment are the key to improve the survival rate and prognosis of ovarian cancer patients. Ultrasound examination has the advantages of repeatability, safety, simple, non-invasive, and reliable. At present, it has been widely used in the clinical diagnosis and follow-up observation of many diseases. Preoperative transvaginal ultrasound in differentiating benign from malignant tumors of ovarian cancer patients is conducive to clinical selection of the appropriate surgical treatment. Ultrasound can not only observe the internal echo, boundary and morphology of the lesion from the perspective of morphology, but also determine the nature of the lesion by observing its hemodynamic characteristics (3, 4). However, two-dimensional transvaginal ultrasound can only provide information about the large vessels of the tumor and macroscopically evaluate the blood supply and distribution inside the lesion, with poor exploration and display results for some microcirculation and small vessels. There are significant differences between the neovascular structure of tumor tissue and normal blood vessel in many aspects such as vessel diameter, basilar membrane walking, vessel diameter, and vascular network morphology (5, 6). With the development of contrast-enhanced ultrasound and related imaging techniques, it is possible to study diseases from the perfusion level of tissue microcirculation, which greatly improves the accuracy of ultrasound diagnosis. Contrast-enhanced ultrasound technology can be used to evaluate the blood vessels of ovarian tumors, improve the sensitivity of ultrasound to the display of deep tissues and microvessels, and has advantages in the detection of small solid ovarian tumors, tumors similar to the acoustic characteristics of ovarian tissues, and low-speed flow ovarian tumors, which is conducive to the detection of ovarian cancer (7–9). In this study, the diagnostic results of transvaginal two-dimensional ultrasound and contrast-enhanced ultrasound were compared in order to clarify the application value of transvaginal two-dimensional ultrasound combined with contrast-enhanced ultrasound in the differential diagnosis of ovarian cancer and provide the basis for clinical diagnosis and treatment of ovarian cancer.

## Data and Methods

### General Information

A total of 100 suspected ovarian cancer patients who were admitted to our hospital from January 2019 to December 2021 were selected as the research subjects, including 62 ovarian cancer patients (ovarian cancer group) and 38 ovarian benign tumor patients (benign group). Inclusion criteria: All patients were confirmed as benign ovarian tumor or ovarian cancer by pathological examination; The patient’s age ≥18 years old; No anti-tumor therapy was performed before the patient was included in the group; There was no history of pelvic surgery before the patient was included in the group. All patients received surgical treatment; All patient had no history of contrast agent allergy. Exclusion criteria: The patients complicated with dysfunction of heart, liver, kidney and other important organs; The patient is combined with malignant tumors in other parts; The patient’s clinical data is missing or incomplete; The patients with mental or consciousness disorders, communication disorders; The patient is a pregnant or lactating woman; The patient had received chemotherapy, radiation and other treatments before entering the group. This study was reviewed and approved by the Hospital Ethics Committee, and the patient or family member informed consent and signed the informed consent form.

### Research Methods

Two-dimensional vaginal ultrasound and contrast-enhanced ultrasound were performed in both groups, and the imaging parameters and characteristics were recorded to determine the diagnostic value. To ensure repeatability and avoid potential differences in device setup, all contrast-enhanced ultrasound examinations and measurements were performed by a senior attending physician.

Two-dimensional ultrasound examination of vagina: The examination instrument was manufactured by GE, USA, and the model was DC-3/DC-3T, with the probe frequency of 7.5 MH_Z_. Before the examination, the patient evacuated the bladder and took the bladder lithotomy position. After the ultrasonic vaginal probe was placed into the vagina, the location, size, and morphology of the ovarian tumor were observed in the two-dimensional mode. Afterwards, the ultrasound mode was turned on to observe and determine the blood flow parameters of the lesion, including arterial pulsatility index (PI), resistance index (RI), end diastolic flow rate (EDV), peak flow rate (PSV), and average flow rate (VM). The average values of the above three parameter measurement cycles were taken.

The instrument was switched to contrast mode, and the patient was asked to remain in position. A rapid bolus of 2.4 mL of SonoVue suspension was injected through the elbow vein, followed by a rapid bolus of 5.0 mL of normal saline for irrigation. The internal timer of the instrument was started synchronously at the time of contrast agent injection to store dynamic images. After the operation, the dynamic images were played back, and the built-in TIC quantitative analysis software was turned on. The parts with more solid tumors and the most abundant blood supply were respectively selected as the observation areas for angiography, with the normal myometrium as the reference. The time-intensity curve (TIC) was automatically generated by the system for quantitative analysis, and the absolute parameters of each TIC were measured and recorded, including initial increase time (AT), maximum peak intensity (IMAX) and peak arrival time (TTP).

### Observation Indicators

The differences of PI, RI, EDV, PSV, VM and other parameters between the two groups as well as the differences of parameters in patients with different grades of ovarian cancer were compared. The contrast-enhanced ultrasound parameters such as AT, TTP and IMAX were recorded.

According to the IOTA standard (10), the tumor was observed under ultrasound for the presence of malignant tumor features (M feature): M1: irregular solid tumor, M2: ascites, M3: at least four papillae, and M4: irregular cystic solid tumor with the largest diameter >10 cm. M5: rich blood flow signals. Benign tumor features (feature B): Bl: single locular cyst, B2: solid component with maximum diameter <0.7 cm. B3: attenuation of sound, B4: multilocular cyst with smooth wall, maximum diameter <10 cm, B5: no blood flow signal. Diagnosis of a benign tumor: Having the features of one or more benign tumors without the features of a malignant tumor. Diagnosis of malignant tumor: Having the features of one or more malignant tumors and not having the features of a benign tumor. Both benign and malignant tumor features are present, or neither is present classified as uncertain type.

Using pathological diagnosis as the gold standard, determine the sensitivity, accuracy, specificity, positive predictive value and negative predictive value of the two-dimensional vaginal ultrasound combined with contrast-enhanced ultrasound in the diagnosis of ovarian cancer.

### Statistical Methods

SPSS22.0 software was used for processing. The measurement data of experimental data were expressed as mean standard deviation $\left(\bar{x}\pm s\right)$, and the enumeration data were expressed as (%). *t* test analysis was used for pairwise comparison of measurement data between groups. The count data were tested by *χ*^2^ test. The test level was *α *=* *0.05, and *p *< 0.05 indicated that the difference was statistically significant.

## Results

### Comparison of General Data Between the Two Groups

There was no significant difference in age, body mass index, course of disease, or lesion diameter between the two groups (*p *> 0.05). As shown in Table 1.

**Table 1:** Comparison of general information of patients between the two groups $\left(n,\text{\%},\bar{x}\pm s\right)$.

Project	Ovarian Cancer Group (*n* = 62)	Benign group (*n* = 38)	*t/χ* ^2^	*p*
Age (years)	45.29 ± 6.07	45.84 ± 5.83	0.446	0.656
Body mass index (kg/m^2^)	21.53 ± 1.19	21.06 ± 1.24	1.887	0.062
Mean course of disease (months)	2.53 ± 0.61	2.61 ± 0.69	0.605	0.546
Menopause			0.315	0.574
Yes	39	26		
No	23	12		
Lesion diameter (cm)	5.13 ± 0.41	5.09 ± 0.37	0.491	0.625

### Pathological Types of Patients in Two Groups

**Figure 1:**
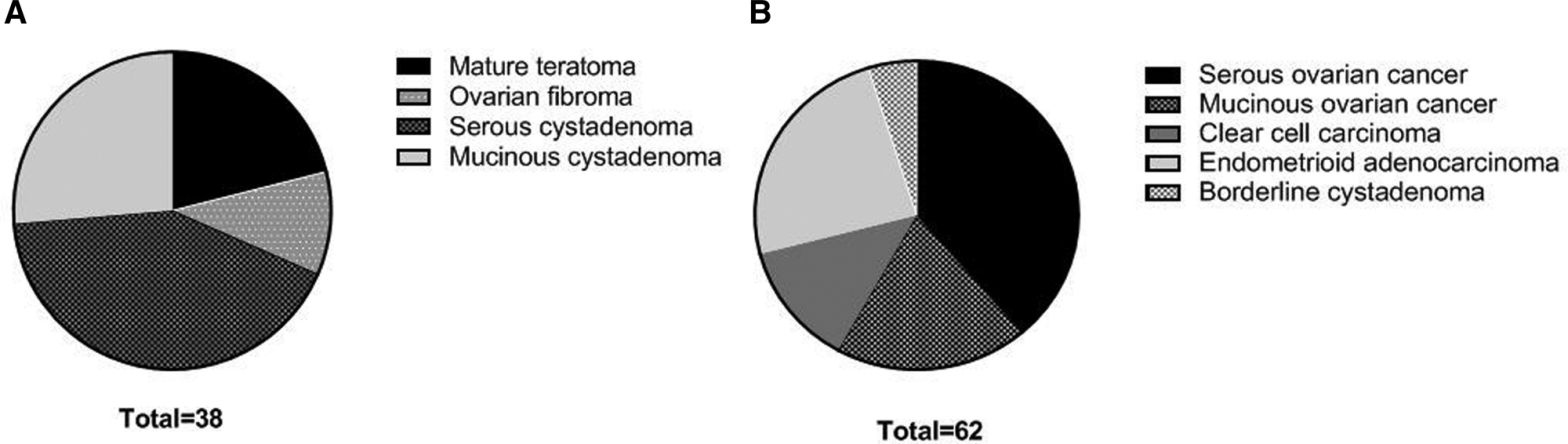
Pathological types of patients in two groups. (**A**) benign ovarian tumors; (**B**) malignant ovarian tumors.

Pathological results showed that 62 patients were malignant ovarian tumors (3 borderline tumors were included in the malignant tumor group due to malignant tendency), and 38 patients were benign ovarian tumors (Figure 1). Among 62 patients of ovarian malignant tumor, 34 patients were in stage I–II and 28 patients were in stage III–IV.

### Comparison of Two-Dimensional Ultrasound Results Between the Two Groups

**Figure 2:**
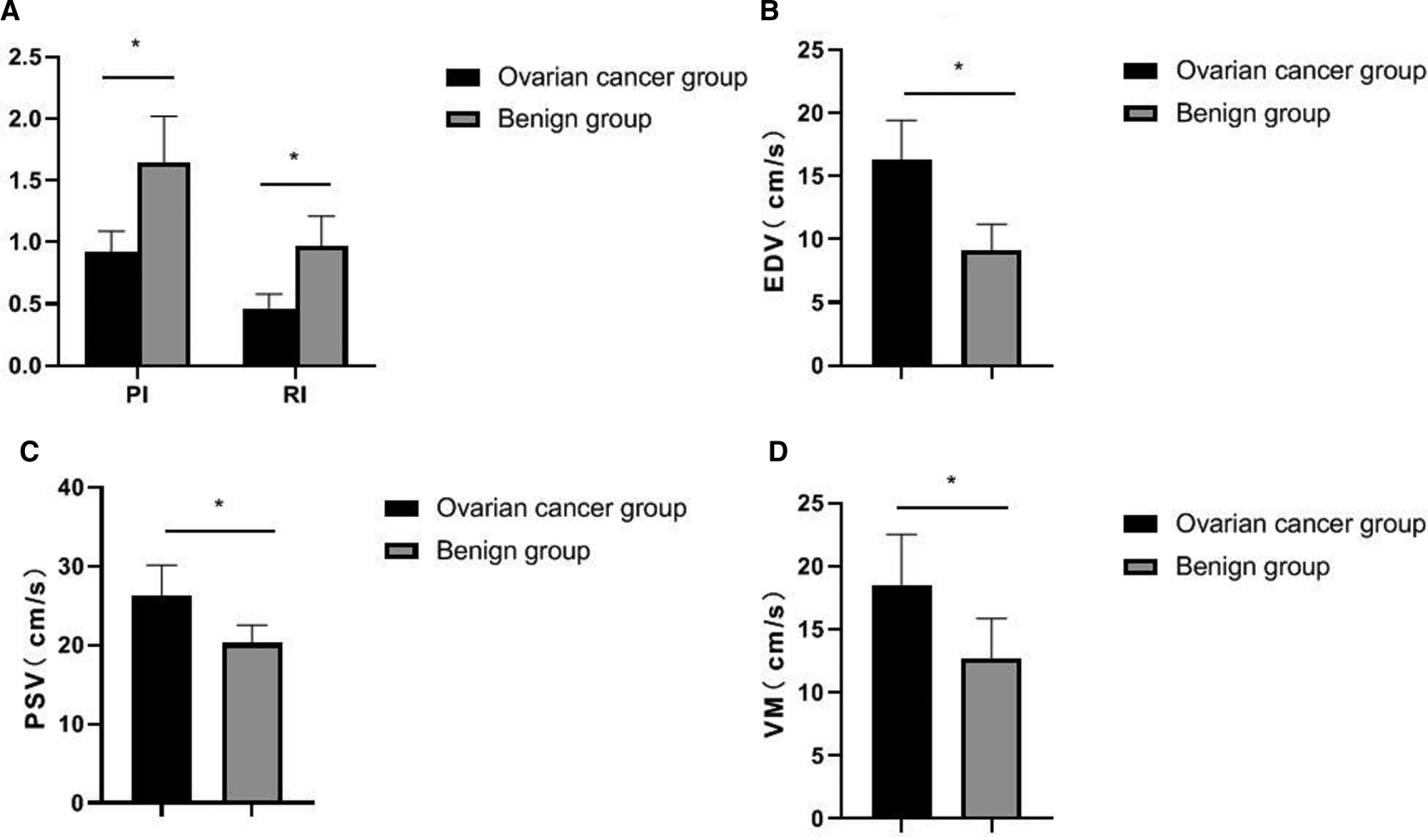
Comparison of two-dimensional ultrasound results between the two groups. (**A**) PI and RI; (**B**) EDV; (**C**) PSV; (**D**) VM.

The PI and RI of the ovarian cancer group were lower than those of the ovarian benign tumor group, and the EDV, PSV and VM were higher than those of the ovarian benign tumor group with statistically significant differences (*p *< 0.05), as shown in Figure 2.

### Comparison of Ultrasound Parameters in Patients with Ovarian Cancer by Different Stages

**Figure 3:**
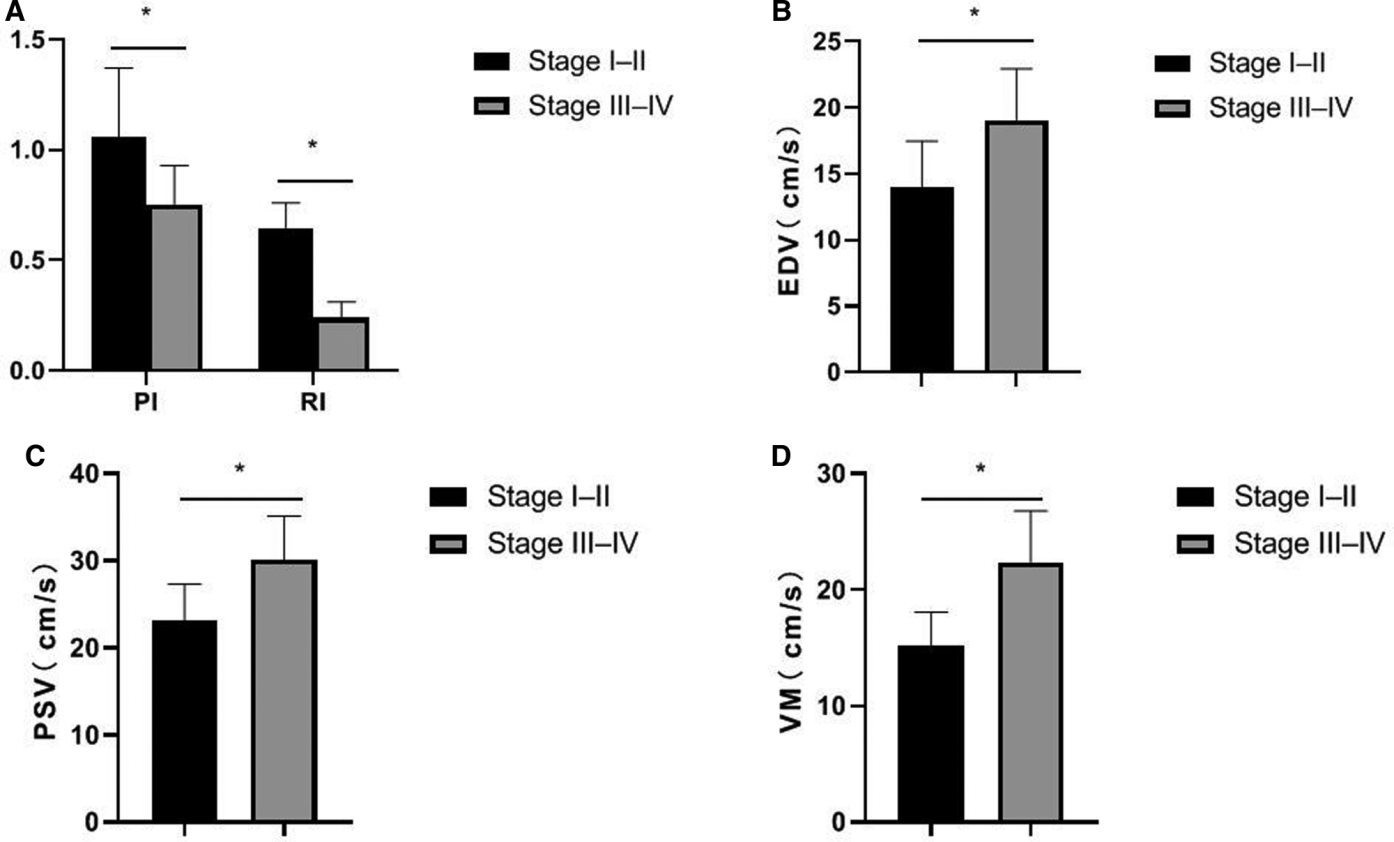
Comparison of ultrasound parameters in patients with ovarian cancer by different stages. (**A**) PI and RI; (**B**) EDV; (**C**) PSV; (**D**) VM.

The PI and RI of the stage I–II patients in the ovarian cancer group were higher than those of the stage III–IV patients, and the EDV, PSV, and VM were lower than those of the stage III–IV patients. The differences were statistically significant (*p *< 0.05), as shown in Figure 3.

### Comparison of Contrast-Enhanced Ultrasound Parameters Between the Two Groups

**Figure 4:**
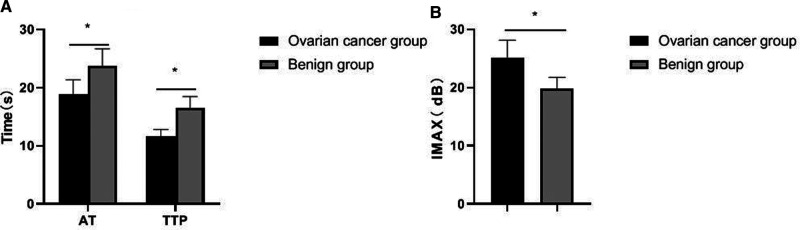
Comparison of contrast-enhanced ultrasound parameters between the two groups. (**A**) AT and TTP; (**B**) IMAX.

The results of contrast-enhanced ultrasound showed that the AT and TTP values in the ovarian cancer group were significantly shorter than those in the benign group, and the peak intensity was significantly higher than that in the benign group, and the differences were statistically significant (*p *< 0.05), as shown in Figure 4.

### Diagnostic Value of Two-Dimensional Ultrasound Combined with Contrast-Enhanced Ultrasound in Ovarian Cancer

**Figure 5:**
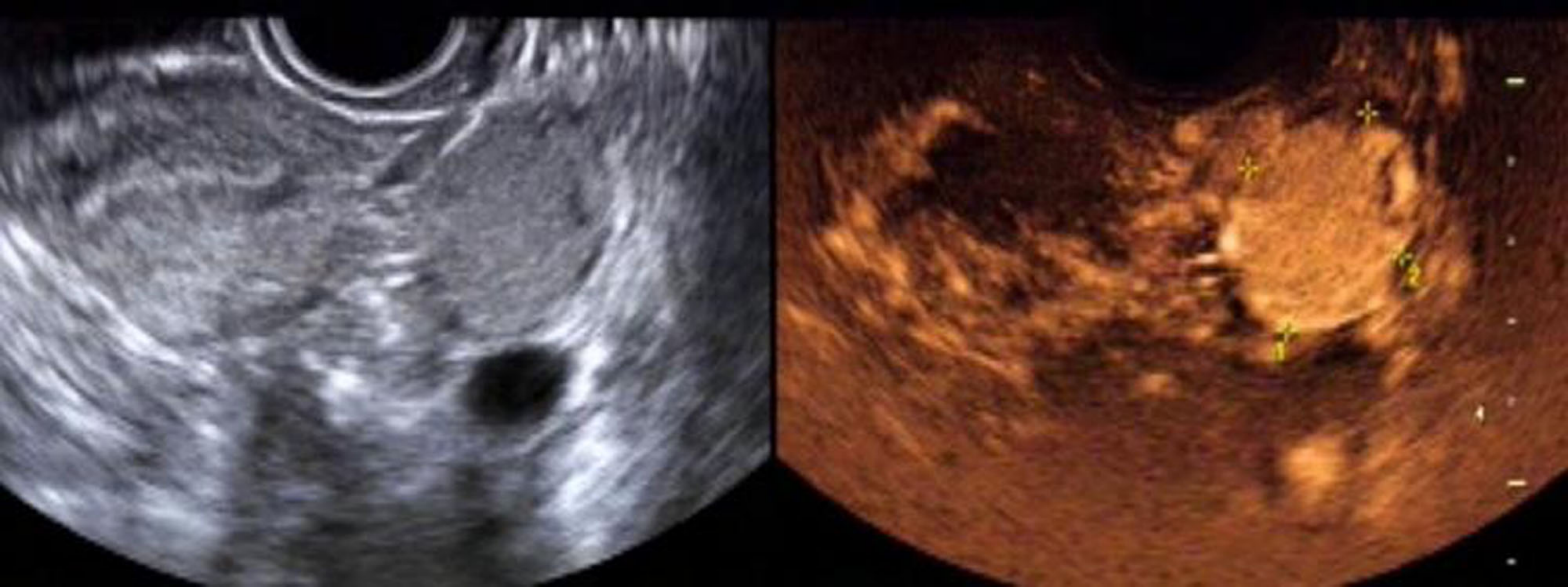
Female patient, 26 years old. Two-dimensional transvaginal ultrasound showed a round hypoecho in the left ovary, measuring about 3.5 cm × 2.4 cm × 3.1 cm, clear and regular in boundary, and uneven in internal echo. On CDFI, blood flow signals in the form of dots and strips were observed. According to contrast-enhanced ultrasound, the lesion in the left ovary was enhanced slightly earlier than that in the myometrium, and rapidly and integrally enhanced from multiple points in the center to the periphery, with the enhancement degree significantly higher than that in the myometrium. The lesion was cleared away from the center to the periphery, presenting as “fast forward and slow reverse”. The capsule echo appeared to be seen from the lesion in the early filling and late resolution.

The sensitivity, specificity, positive predictive value, negative predictive value and accuracy of two-dimensional ultrasound combined with contrast-enhanced ultrasound in the diagnosis of ovarian cancer were high, 95.16%(59/62), 86.84%(33/38), 92.19%(59/64), 91.67%(33/36) and 92.00%(92/100), respectively. As shown in Table 2. The image of a typical case can be seen in Figure 5.

**Table 2:** Comparison of diagnostic results between two-dimensional ultrasound and contrast-enhanced ultrasound.

Pathology	Number	Two-dimensional ultrasound		Contrast-enhanced ultrasound		Combined diagnosis	
		Malignant	Benign	Malignant	Benign	Malignant	Benign
Malignant ovarian tumors	62	54	8	56	6	59	3
Benign ovarian tumors	38	16	22	7	31	5	33
Total	100	70	30	63	37	64	36

## Discussion

The occurrence of ovarian cancer is closely related to genetic, environmental and other factors. The prevalence of ovarian cancer has an increasing trend in China and abroad, and has become one of the reproductive tract malignant tumors that seriously affect the health of women. At the same time, ovarian cancer and benign ovarian lesions are common gynecological diseases, and their early clinical manifestations are extremely similar. However, in clinic, the treatment methods and prognosis are completely different, so the early and correct differential diagnosis of ovarian cancer is of great significance for the prognosis of patients (11–13). Transvaginal two-dimensional ultrasound is currently a common screening method for malignant tumors. The distribution and nature of tumors can be observed by two-dimensional ultrasound, and the nature of a mass can be comprehensively judged by the characteristics of whether the mass has an envelope or not (14).

Angiogenesis of ovarian tumors is the pathological basis for the differentiation of benign and malignant ovarian tumors. The arterial system and venous system of normal ovaries are generally parallel and distributed in a tendril shape. Ovarian cancer grows faster and generates more new blood vessels, which constitutes a larger pressure difference, and there will be different levels of blood flow signals in or around the essence of the lesion (15). Under the ultrasound examination, malignant tumor mass mainly manifests as a large number of new blood vessels. The new blood vessel blood flow is characterized by low resistance and high flow velocity, and often accompanied by abnormal shape and arteriovenous fistula. Therefore, observing the new blood vessels can judge the benign and malignant tumor. In contrast, the blood flow signals in or around benign ovarian tumors such as fibromas are not rich, and the RI is high, so there is usually no obvious blood flow signal (16). In this study, the criteria for the diagnosis of ovarian cancer by contrast-enhanced ultrasound were as follows: After contrast-enhanced ultrasound was performed, bleeding and necrotic areas were observed, the lesion morphology was irregular, and the contrast process showed fast forward and fast reverse. There was local filling defect of contrast medium in the lesion and its distribution was not uniform. At the time of clearance, there was local manifestation of contrast medium retention (17, 18). In this study, the PI and RI of the ovarian cancer group were lower than those of the ovarian benign tumor group, and the EDV, PSV, and VM were higher than those of the ovarian benign tumor group. In addition, the PI and RI of patients in stage I–II of the ovarian cancer group were higher than those in stage III–IV patients, and the EDV, PSV and VM were lower than those in stage III–IV patients. It indicated that two-dimensional transvaginal ultrasound was helpful to distinguish benign and malignant ovarian tumors, and had certain clinical value in judging the progression and staging of ovarian cancer.

The development of contrast-enhanced ultrasound and related technologies has improved the ability of ultrasound to detect lesions and qualitative diagnosis. Contrast-enhanced ultrasound is based on conventional two-dimensional ultrasound examination. The perfusion of contrast agent into the microvessels can further enhance the visualization of tissues and organs, fully displaying the microvessels in the mass, helping to find the basal part of the cancer focus. At the same time, the boundary between the focus and the normal myometrium is clear, which provides a good prerequisite for further judgment on the activity of the focus in clinical practice (19–21). The contrast-enhanced ultrasound examination results in this study showed that the AT and TTP values in the ovarian cancer group were significantly shorter than those in the benign group, and the peak intensity was significantly higher than that in the benign group. This is because tumor growth and infiltration are closely related to angiogenesis. In particular, one of the typical characteristics of malignant tumors is the large amount of neovascularization. Along with the growth of new blood vessels, the tumors also grow and metastasize rapidly. The characteristics of neovascularization are that the tube wall is relatively thin, without smooth muscle, and it is only composed of endothelial cells, so its elasticity is poor, and its distribution is also relatively disorder, and the tube lumen is relatively thickened. Therefore, if a large number of new blood vessels are found in the lesion, it often indicates that the lesion is malignant (22, 23).

There are many types of ovarian tumors, and their pathological structures are relatively complex, with different forms and properties. Many ovarian tumors have the same disease map or the same disease with different maps, which brings some difficulties to the differential diagnosis by ultrasound. Therefore, how to improve the coincidence rate of ultrasonic examination and pathological diagnosis is the key to the differential diagnosis of benign and malignant ovarian tumors (24, 25). In this study, the sensitivity, specificity, positive predictive value, negative predictive value and accuracy of two-dimensional ultrasound combined with contrast-enhanced ultrasound in the diagnosis of ovarian cancer were improved, 95.16%, 86.84%, 92.19%, 91.67% and 92.00%, respectively.

The main reasons for misdiagnosis of two-dimensional ultrasound were as follows: the lesion size was small, the ultrasonographic manifestations of benign and malignant ovarian tumors were similar, and the difference in color blood flow signals and RI of blood flow in lesions between benign and malignant ovarian tumors was not significant (26). In this study, one case (stage I ovarian cancer) underwent two-dimensional ultrasound examination, and it was considered as a subserosal myoma with cystic degeneration. However, 11 s after injection of contrast agent, the thick-walled solid area around the lesion showed rapid enhancement, which was the feature of high-perfusion malignant tumor, and it was finally diagnosed as malignant. Contrast-enhanced ultrasound can fully display the structure, number, and course of blood vessels inside the tumor by injecting contrast agent to enhance the blood backscatter signal, thus improving the finalize the diagnosis of the lesion. The main reasons for misdiagnosis of contrast-enhanced ultrasound examination are that the contrast observation object is the microvessels in and around the ovarian mass, and the cross and overlap of enhancement patterns of some benign and malignant masses are easy to cause misdiagnosis (27). In this study, two inflammatory masses were misdiagnosed as ovarian cancer by contrast-enhanced ultrasound. It was possible that the blood vessels were dilated under the stimulation of inflammatory factors, which increased the local blood flow and accelerated its flow velocity. The masses were rapid and high enhancement mode during contrast-enhanced ultrasound, which eventually led to the misdiagnosis.

This study has certain limitations. The sample size included in the study is small, and further sample size accumulation is required. Besides, two-dimensional ultrasound and contrast-enhanced ultrasound are the combination of image technology and contrast technology, which have certain limitations in the process of image acquisition, reconstruction and processing, and need to be further studied.

In summary, contrast-enhanced ultrasound to a certain extent makes up for the deficiencies of conventional ultrasound, is conducive to the detection of early ovarian cancer, and can be used for the differential diagnosis of small ovarian tumors with difficulties in two-dimensional ultrasound diagnosis. Two-dimensional ultrasound combined with contrast-enhanced ultrasound can effectively improve the detection rate and differential diagnosis value of ovarian cancer, which is of great significance in the early diagnosis and differentiation of ovarian cancer.

## Data Availability

The original contributions presented in the study are included in the article/Supplementary Material, further inquiries can be directed to the corresponding author/s.
